# An Evaluation of Food Allergy Management Practices in a Sample of Canadian and American Schools

**DOI:** 10.3390/nu17121971

**Published:** 2025-06-10

**Authors:** April Quill, Michael A. Golding, Lisa M. Bartnikas, Jennifer L. P. Protudjer

**Affiliations:** 1 Department of Statistics, University of Manitoba, Winnipeg, MB R3T 2M5, Canada; quilla@myumanitoba.ca; 2 Children’s Hospital Research Institute of Manitoba, Winnipeg, MB R3E 3P4, Canada; michael.golding@umanitoba.ca; 3 Department of Pediatrics and Child Health, Max Rady College of Medicine, Rady Faculty of Health Sciences, University of Manitoba, Winnipeg, MB R3E 0W2, Canada; 4 Division of Immunology, Department of Medicine, Boston Children’s Hospital, Boston, MA 02115, USA; lisa.bartnikas@childrens.harvard.edu; 5 Harvard Medical School, Harvard University, Boston, MA 02115, USA; 6 Department of Food and Human Nutritional Sciences, Faculty of Agricultural and Food Sciences, University of Manitoba, Winnipeg, MB R3T 2N2, Canada; 7 Institute of Environmental Medicine, Karolinska Institutet, 17177 Stockholm, Sweden

**Keywords:** allergic reaction, anaphylaxis, children, schools management

## Abstract

**Background:** Children, including the estimated 7% with food allergy, spend most of their waking hours in school. Variations in school-based food allergy (FA) practices exist. We aimed to examine differences in FA management practices across schools in Canada and the United States (US). **Methods:** Parents of children with Immunoglobulin E (IgE)-mediated FA were recruited through social media to complete a survey evaluating the schools’ stock epinephrine, epinephrine storage locations, school type, and location. Data were described, analyzed using logistic and linear regressions, and then reported as odds ratios (ORs) and standardized coefficients (b), respectively, with corresponding 95% confidence intervals (95%CIs) and *p* < 0.05. This study was approved by the University of Manitoba Health Research Ethics Board. **Results:** Overall, 177 participants (14% [26/177] Canada, 86% [151/177] US) were included. Children were, on average, 4.92 ± 3.12 years and were commonly but not mutually exclusively allergic to tree nuts (50% Canada; 40% US) and peanuts (33% Canada; 29% US). Compared to US parents, Canadian parents were more likely to report epinephrine self-carriage by their children (OR = 4.58; 95%CI = 1.67–12.59). Parents with children age > 5 years were more likely to report epinephrine self-carriage by their children (OR = 3.70; 95%CI = 1.38–9.93) but less likely to report that their children’s school had an allergen-friendly zone (OR = 0.25; 95%CI = 0.06–0.99). Compared to US parents, Canadian parents were more likely to report their child’s school had anaphylaxis management policies (OR = 8.98; 95%CI = 1.11–72.42). **Conclusions:** Significant in-school FA management differences exist between countries. These findings stress the need for consistent policies and practices to ensure effective care.

## 1. Introduction

Approximately 7% of children in North America have Immunoglobulin E (IgE)-mediated food allergy, with children under 5 years old being most likely to be affected [1,2,3]. While those with food allergy can live much of their lives free from symptoms, exposure to the trigger food can cause acute reactions. The severity of reactions can range widely, from mild (e.g., hives) to potentially life-threatening, multisystem symptoms or anaphylaxis, for which children need to be treated immediately with intramuscular epinephrine, commonly delivered via an epinephrine autoinjector (EAI). Given that children spend most of their waking hours in school, it is not surprising that approximately 10–20% of food allergic reactions occur at school and childcare centers [4,5,6]. As such, there is an obvious need for policies and practices that aim to mitigate the risk of reactions and procedures to follow when such reactions occur while children are at school or childcare centers.

In both Canada and the United States of America (US), national guidelines are available to aid government officials and school administrators in the development of policies and procedures pertaining to the management of food allergy in school [7,8]. Despite this, only some regional governments and school districts have policies in place, and what policies do exist are often highly variable across jurisdictions [9,10,11,12]. Such variability includes but is not limited to food allergy preparedness, including the student’s ability to self-carry and/or self-administer their own EAI; training and authorization for school personnel to administer EAI; staff training; restrictions on what foods can be brought to schools or, if permitted, where students can eat known allergens; and food allergy and anaphylaxis action plans [13,14].

In an attempt to address this variability, an international consortium of researchers and stakeholders reviewed the available evidence on the management of food allergy in school and childcare settings with the aim of creating the first international set of guidelines on managing food allergy at school [4]. Based on the available evidence, the panel recommended that schools and childcare centers implement food allergy preventive training, personal allergy action plans, and protocols for managing reactions in students without plans [4]. They also recommended that schools carry unassigned (or stock) epinephrine based on its cost-effectiveness and its ability to improve access to the medication. In contrast, the panel did not recommend that epinephrine be administered prior to the development of symptoms due to a lack of research on its efficacy and its potential for harm. They also did not recommend the use of site-wide food prohibitions or allergen-restricted zones, unless the students were incapable of self-management due to being very young in age. Based on their review of the literature, the panel concluded that the evidence supporting the efficacy of restrictions on food allergens was not conclusive enough to outweigh its potential limitations [4].

As the above are recommendations rather than requirements, the extent to which they have been adopted by schools and childcare centers in Canada and the US is unclear. To address this knowledge gap, we aimed to explore food allergy management practices in schools across Canada and the US, including the availability of stock epinephrine, epinephrine storage locations, the use of anaphylaxis protocols, site-wide food prohibitions and allergen-restricted zones. Moreover, we explored such management practices with consideration to school type and the child’s age.

## 2. Methods

### 2.1. Study Design and Participant Recruitment

This analysis is based on data from an online, cross-sectional study involving Canadian and US parents/caregivers (hereafter “parents”) who were recruited between February and March 2024 through social media advertisements on food allergy-focused pages and groups. Advertisements for this study included a link to an online consent disclosure statement, which provided a complete overview of this study’s eligibility requirements, procedures, and privacy safeguards. Parents were eligible to participate if their child had an IgE-mediated food allergy diagnosed by a physician and the child was enrolled in pre-school (i.e., children not old enough to start formal school but who attended daycare or childcare, and/or age-appropriate learning programs outside of the home), elementary school (Kindergarten to Grade 5), middle school (Grades 6–8), or high school (Grades 9–12). As the survey was only available in English, parents were required to self-determine if they were comfortable completing an English-language questionnaire. Parents of children who were home-schooled were not eligible. If, after reading the consent disclosure statement, parents were interested and eligible in participating, they were asked to advance to the survey package by clicking the next page button. The survey was completed anonymously and contained questions regarding their understanding of their child’s school’s food allergy management. All data was collected and managed using the REDCap survey software. Parents who completed the survey could opt to receive a CAD 5 e-gift card honorarium; those who opted to receive this were redirected to a separate link where they entered their name and contact details. This study was approved by the University of Manitoba Health Research Ethics Board. As data were collected as part of an anonymous survey and participants were not required to provide any personal identifiers (e.g., name and date of birth), participants responded to the consent disclosure statement, per our institutional ethical approval.

### 2.2. Measures

Parents were asked to complete an ad hoc survey containing questions related to household demographics and their child’s school’s food allergy management practices. Demographic questions included those related to age, country of residence (i.e., Canada and the US), highest level of parental education, and household income (in Canadian dollars [CAD] or US dollars [USD], depending on country of residence). When reporting income, parents could select from one of eight categories (<$20,000; $20,000–$34,999; $35,000–$49,999; $50,000–$74,999; $75,000–$99,999; $100,000–$149,999; $150,000–$199,999; $200,000 or more). We converted Canadian dollars (CAD) to US dollars (USD) using exchange rates tied to the specific timestamps reported by participants, ensuring the correct rate was applied. Parents also self-reported the race of both the responding parent and the index child per the US census guidelines, which were adapted for Canada. Racial categories roughly corresponded to those found in the US census, with some changes aimed at better reflecting the Canadian context. For instance, “Indigenous” was used rather than American Indian in order to be applicable to both Canadian and US participants. If participants did not identify with one of the pre-determined racial categories, they could select the “other” option, which provided them with the opportunity to indicate their race using a free response box. As part of the demographic questions, respondents were also asked to report the number and type of the index child’s food allergies (closed-ended list with multiple choices permitted, including cow’s milk, soy, egg, wheat, peanuts, tree nuts, fish, shellfish, sesame, and other).

After completing the demographic items, parents were asked to complete a number of questions about their child’s pre-school or school. These included questions about the school type (public vs. private); school location (self-described urban/suburban area or rural/remote area); and the child’s age.

As part of these questions, parents were asked to report whether the school exercised restrictions on certain food allergens. Primary outcomes related to food allergy management included the availability of stock epinephrine; epinephrine storage locations (closed-ended options with multiple choices permitted included classroom, health office, main office, and student carries their own epinephrine); the use of anaphylaxis action plans; site-wide food prohibitions; and allergen-restricted zones (all of which had binary responses of yes or no [collapsed from no to unsure]).

Parents were also asked to rank both how comfortable they were with the school’s food allergy management policies and how well they perceived these policies were enforced. In both instances, the scale was 1–10, with higher scores indicating a greater level of comfort/enforcement (please see Supplementary Materials for the complete survey).

### 2.3. Data Analyses

Descriptive analyses (n/N, means, standard deviations [SD], and percentages [%]) were used to describe in-school food allergy management practices and parents’ comfort with these practices and their perceived level of enforcement. As part of descriptive analyses, we also checked for missing data, a process that resulted in the identification of missing data in multiple variables, with the amount ranging from 0 to 34%. To maintain power and minimize bias, 100 datasets were created using multiple imputation. Following imputation, a series of binary logistic regression analyses were used to assess differences in allergy management practices in schools across Canada and the US, school types (public vs. private), and the child’s age. Multiple linear regression was used to predict differences in parents’ comfort with their child’s school’s food allergy policies and their perceived level of enforcement across Canada and the US, school types (public vs. private), and the child’s age. Age was expressed categorically (5 years and under vs. greater than 5) in each of the regression analyses for ease of interpretation. Each of the logistic and linear regression analyses was also adjusted for household income in USD. Multiple linear and logistic regressions were reported as unstandardized regression coefficients (b) and odds ratios (ORs), respectively, with 95% confidence intervals (95%CIs) and *p*-values. An alpha value of *p* < 0.05 was set a priori.

## 3. Results

### 3.1. Demographics

**Figure 1:**
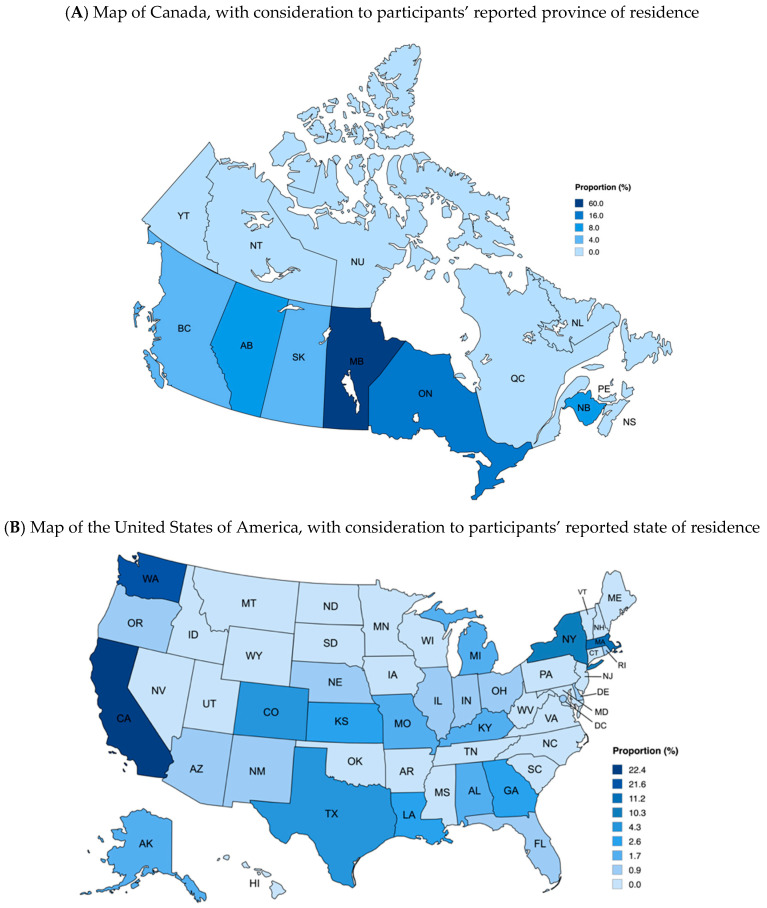
Heat maps of participants’ reported provinces (**A**) and states (**B**) of residence. Maps created using mapchart.net.

In total, 177 participants were recruited, with 14% (26/177) from Canada and 86% (151/177) from the US. In Canada, participants reported residing in Manitoba (60%), Ontario (16%), New Brunswick (8%), and Alberta (8%) (see Figure 1A). The majority of participants from the US resided in the West (53%), followed by Northeast (22%), South (16%), and Midwest regions (9%) (see Figure 1B). Most parents identified as women (88% Canada; 64% US). All educational levels were reported, with the most common level among Canadian parents being a graduate school degree (40%), while US parents reported having a junior or community college degree (39%). Children were approximately 5 years old, on average (total sample = 4.91 [SD = 3.11]; Canada = 6.25 [SD = 3.32]; US = 4.66 [SD = 3.02]), and most of the sampled children were males (60% Canada; 58% US). Canadian parents most commonly reported that their child attended elementary school (40%), whereas most US parents reported their child attended a childcare center, pre-school, or pre-kindergarten (55%). The majority of children attended public school (60% Canada; 66% US) and were most commonly allergic to tree nuts (50% Canada; 40% US) and peanuts (33% Canada; 29% US). Children were predominantly White (76% Canada; 87% US), with smaller numbers of Black or African American (4% Canada; 7% US), Asian (4% Canada; 3% US), and multi-race (16% Canada; 1% US) individuals represented. Household income categories were all approximately comparably represented (see Table 1).

### 3.2. Parental Descriptors of Their Child’s School’s Policies

Most parents reported their child’s school had stock epinephrine available for emergency use (59% Canada; 83% US) and had a protocol in place for anaphylaxis management (91% Canada; 65% US). Parents also reported that their children were prohibited from bringing certain food allergens to their school or childcare center (83% Canada; 74% US). Additionally, most parents reported that certain food allergens were restricted in specific areas of their child’s school (68% Canada; 70% US). About 62% of US parents reported that their child’s school had allergen-friendly zones in the cafeteria compared to 30% of Canadian parents. Moreover, Canadian parents reported their mean level of comfort with their child’s school’s food allergy policies and their child’s school’s food allergy policy enforcement as 6.77 (SD = 2.10) and 7.35 (SD = 1.98), respectively. By comparison, US parents had means of 6.13 (SD = 2.08) and 6.22 (SD = 2.15), respectively (see Table 2).

### 3.3. Food Allergy Management Policies Across Countries, School Type, and Child Ages

Compared to US parents, Canadian parents were more likely to report that their children carried their EAI on their person (OR = 4.58; 95%CI = 1.67–12.59; Table 3). In addition to country of residence, children over the age of 5 years were also more likely to carry their own epinephrine relative to children less than 5 years (OR = 3.70; 95%CI = 1.38–9.93; Table 3). Conversely, compared to children under the age of 5 years, children older than 5 years were less likely to have their EAI stored in the main office (OR = 0.31; 95%CI = 0.10–0.99; Table 3). Compared to US parents, Canadian parents were more likely to report their child’s school had anaphylaxis management policies in place (OR = 8.98; 95%CI = 1.11–72.42; Table 4). The results also revealed that the likelihood of parents reporting allergen-free zones in specific areas of the school was also lower among children over the age of 5 years (OR = 0.25; 95%CI = 0.06–0.99; Table 5) compared to children 5 years and under. In contrast, neither age nor the other included variables were found to significantly predict the likelihood of school-wide restrictions on students bringing certain food allergens from the home or allergen-free zones in the school’s cafeteria.

None of the included variables significantly predicted the odds that the child’s school had stock epinephrine or if the children stored their epinephrine in the classroom or health office (Table 6).

### 3.4. Perceived Enforcement and Comfort with School’s Food Allergy Management Policies Across Countries, School Type, and Child Age

School type (i.e., public vs. private) was significantly associated with parents’ comfort with the allergy policies at their child’s school. Specifically, parents with children attending private school reported being significantly more comfortable with the school’s allergy policies compared to children attending public school (b = 1.07; 95%CI = 0.30–1.84; Table 7). Additionally, compared to US parents, Canadian parents reported that their children’s school was more likely to enforce food allergy policies (b = 1.01; 95%CI = 0.13–1.89; Table 7).

## 4. Discussion

Our study explored the knowledge gap of food allergy management practices in public and private schools across Canada and the US. We identified that most parents reported that their child’s school had both stock epinephrine and anaphylaxis protocols in place, although it is interesting to note that more parents reported the former than the latter. Epinephrine self-carriage was most commonly reported in the older age group and in Canada vs. the US. Additionally, there were lower odds of epinephrine being stored in the main office for the older age group, possibly related to increased rates of EAI self-carriage in this age group. Allergy-restricted zones were reported most commonly in the youngest age group. Parental comfort with their child’s school’s food allergy policies and their perceived level of enforcement were moderate, but with notably greater comfort and perceived enforcement in private vs. public schools and Canadian vs. US schools.

Epinephrine is the only known drug that can halt or reverse anaphylaxis [15]. Regular training, coupled with knowledge of a child’s anaphylaxis action plan (including where epinephrine is stored and the child’s known food allergies), is paramount. Such training, delivered by food allergy experts, is encouraged to be part of professional education. Yet, such training is seemingly under-delivered. In a study from Washington State, US, most school nurses (94%), only slightly more than half (59%) of teachers, and only half (51%) of food service staff reported previous food allergy training [16]. It follows logically that, in schools in which a full-time school nurse is available, that person may be the one most likely to assume care for a child experiencing an allergic reaction. However, not all schools have a full-time nurse on site. Indeed, a qualitative study from Winnipeg, Canada, provides evidence that teachers receive training from a nurse prior to the start of the academic year [12]. However, thereafter, teachers were often left to rely on their own knowledge about food allergy and spoke of “little standardization” of policies between school types and even across and within schools from the same regulatory authorities [12].

The purchase of EAIs can come with high out-of-pocket costs for families [17,18]. While a majority of patients prescribed an EAI do indeed fill them, reasons for not filling them range from never previously needing an EAI to cost. In an era when inflation is outpacing wage increases in many jurisdictions, there is a real possibility that families may need to make hard financial choices. Although EAIs are generally less expensive in Canada compared to the US, they can still be cost-prohibitive for some families [18]. Similarly, in the US, out-of-pocket costs for EAIs fluctuate significantly, even among those with private insurance [19]. On account of potentially more limited access, stock epinephrine should not be considered equivalent to self-carriage. However, stock epinephrine may be of value for those who are not financially able to procure their own EAI or for students who have forgotten their own device. Notably, stock epinephrine, compared to self-carriage, is cost-effective based on modeling from the US [20].

Parents of children with food allergy often assume an advocacy role, particularly when the child is still young. Yet, we identified that parental comfort with their child’s school’s food allergy policies and their perceived level of enforcement were moderate. Higher levels of comfort were reported by parents whose children were in private vs. public schools. While our study was not designed to explore these differences, it is reasonable to hypothesize that such differences may in part result from school and class size, and ease of gaining access to speak with school leadership. Reasons for the differences between Canadian vs. US schools are less easily theorized and warrant further investigation.

The present study was based on data collected in early 2024, that is, in the aftermath of the acute phases of the COVID-19 pandemic but still when knowledge of related policies was familiar. During the pandemic, food bans were not recommended [21]. Indeed, even prior to the pandemic, they were discouraged, although designated allergy-free tables appear to be associated with lower rates of epinephrine administration and thus may be appropriate in well-defined circumstances, such as amongst infants and toddlers, or if physical or cognitive impairments limit self-management of food allergy [4,22]. This topic is likely to remain controversial for the foreseeable future. Moreover, there are almost certainly other variables that need to be further explored and subsequently tailored to any particular school.

We acknowledge the limitations of our study. The sample size for Canada was relatively small. Although it is not clear why US citizens participated in greater numbers, it may simply reflect their disproportionately large population or differential use of social media platforms used for study recruitment. It should also be mentioned that there was an unequal distribution of participants within each country. In Canada, more than half of the participants were from Manitoba and other western provinces, and within the US sample, there was greater representation of participants residing in the Western region, with relatively fewer participants from the South and Midwest regions. It is not clear why there was a disproportionate number of participants from particular regions, but it is reasonable to assume that it reflects the use of convenience sampling. What is more clear, however, is that the disproportionate number of participants from particular regions may limit our ability to make broad generalizations about Canadian and US food allergy management practices in schools. In light of this limitation, future studies focused on the management of food allergy in schools should aim to recruit a larger and more geographically diverse sample. Another limitation is the sample’s relative paucity of participants from non-White racial groups. This lack of diversity may make it challenging to generalize the findings to non-White populations. In light of this limitation, we recommend that future research investigating food allergy management practices in schools put specific measures in place aimed at recruiting a racially and ethnically diverse sample. It should also be mentioned that the sample included a disproportionate number of pre-school-aged children. Some of the overrepresentation of younger children may stem from the fact that caregivers were unable to report on multiple children. Therefore, it is possible that caregivers who had multiple children simply completed the questionnaires in regard to their youngest child. While the practice of having parents report on only one child was aimed at limiting participant burden, it does, unfortunately, limit the range of available information and our understanding of the demographics of the sample, to some degree. Parents’ descriptions of their child’s school’s food allergy management policies may differ from the actual policies of the school. While it would have been preferable to validate parent reports with the actual policies of the schools, conducting this would have required far more time and resources than were available for the current study. It is possible that parents of children attending schools with certain characteristics were more or less likely to participate in this study. Additionally, since we did not collect data on specific school names attended by participants, it is possible that some schools were represented multiple times, which could bias the results.

The strengths of this study are notable. Herein, we provided novel information on how food allergy management practices differ across Canada and the US, school types (private vs. public), and the age of the child. To our knowledge, this is the first study to compare these practices between the two countries. Our recruitment strategy, while rooted in social media, was specifically targeted to those affected by food allergy. Thus, we are confident that our results do indeed reflect what parents believe to be happening in schools.

## 5. Conclusions

Looking forward, a wider, more in-depth investigation employing a mixed-methods design may help glean greater insight, and in turn, guide evidence-based, parent-informed, food allergy management practices that are tailored to specific settings.

In summary, while stock epinephrine and epinephrine storage locations are similar between countries, differences were identified among older vs. younger children. Best practices and ongoing knowledge translation efforts are essential to ensure that epinephrine is consistently available in known, easily accessible locations to immediately treat anaphylaxis in schools.

## Figures and Tables

**Table 1:** Demographic characteristics of participating parents, their children, and their households.

	Canada		United States	
	n	%	n	%
**Parent of child with food allergy (N = 177)**				
Parent’s gender				
Woman	22	88.0	95	64.2
Man	3	12.0	53	35.8
Non-binary	0	0.0	0	0.0
Parent’s education				
High school	1	4.0	10	6.8
GED or high school equivalency	1	4.0	39	26.4
Junior/community college degree	4	16.0	58	39.2
Undergraduate degree	9	36.0	15	10.1
Graduate school degree	10	40.0	26	17.6
**Child with food allergy**				
Child’s age in years				
≤5 years	13	68.4	89	84.0
>5 years	6	31.6	17	16.0
Child’s gender				
Girl	10	40.0	62	41.9
Boy	15	60.0	86	58.1
Non-binary	0	0.0	0	0.0
Child’s race				
Black/African American	1	4.0	11	7.4
Hispanic or Asian	1	4.0	6	4.1
White	19	76.0	129	87.2
Multi-Race	4	16.0	2	1.4
Indigenous	0	0.0	0	0.0
Child’s current grade level				
Pre-school	9	36.0	79	54.5
Elementary school	10	40.0	56	38.6
Middle school	3	12.0	4	2.8
High school	3	12.0	6	4.1
Child’s school type				
Public	15	60.0	78	66.1
Private	10	40.0	40	33.9
**Household characteristics**				
Household income (CAD/USD)				
<$34,999	9	5.3	41	24.0
$35,000–$49,999	38	22.2	44	25.7
$50,000–$74,999	38	22.2	16	9.4
$75,000–$99,999	16	9.4	11	6.4
$100,000–$149,999	11	6.4	8	4.7
$150,000–$199,999	8	4.7	37	21.6
>$200,000	51	29.8	14	8.2
Type of residence				
Rural/remote	1	4.0	32	25.6
Urban/suburban	24	96.0	93	74.4

Abbreviations: GED, general educational development; CAD, Canadian dollars; USD, United States dollars.

**Table 2:** Parental descriptions of food allergy policies.

	Canada			United States		
	n	%	Mean (SD)	n	%	Mean (SD)
Parent’s comfort with the school’s current food allergy protocols (1–10 scale)			6.8 (2.1)			6.1 (2.1)
Perceived enforcement of the school’s food allergy protocols (1–10 scale)			7.3 (2.0)			6.2 (2.1)
Food allergens restricted from being sent to school						
No	4	17.4		29	25.7	
Yes	19	82.6		84	74.3	
Parent-reports of allergen free zones						
Cafeteria	6	30.0		68	62.4	
Specific classrooms	15	68.2		75	70.1	
Anaphylaxis management protocol						
No	2	9.5		46	35.4	
Yes	19	90.5		84	64.6	
Stock epinephrine available						
No	7	41.2		17	17.2	
Yes	10	58.8		82	82.8	
Location of epinephrine storage						
Classroom	10	38.5		13	8.6	
Health office	4	15.4		71	47.0	
Main office	7	26.9		47	31.1	
Student’s self-carriage						
No	14	53.9		126	83.4	
Yes	12	46.2		25	16.6	

Abbreviations: SD, standard deviation.

**Table 3:** Logistic regressions predicting epinephrine storage in the classroom, health office, main office, and students’ self-carriage.

	OR	SE	95%CI	*p*-Value
DepVar: Classroom storage				
Countries				
United States	Ref	-	-	-
Canada	1.41	0.88	0.42; 4.78	0.58
School Type				
Public	Ref	-	-	-
Private	0.95	0.49	0.34; 2.60	0.92
Child age				
≤5 years	Ref	-	-	-
>5 years	1.25	0.78	0.36; 4.27	0.73
DepVar: Health office storage				
Countries				
United States	Ref	-	-	-
Canada	0.33	0.19	0.11; 1.00	0.05
School Type				
Public	Ref	-	-	-
Private	0.69	0.29	0.31; 1.57	0.38
Child age				
≤5 years	Ref	-	-	-
>5 years	1.31	0.70	0.46; 3.75	0.62
DepVar: Main office storage				
Countries				
United States	Ref	-	-	-
Canada	1.25	0.65	0.45; 3.48	0.67
School Type				
Public	Ref	-	-	-
Private	1.89	0.74	0.88; 4.06	0.10
Child age				
≤5 years	Ref	-	-	-
>5 years	0.31	0.18	0.10; 0.99	0.048
DepVar: Student’s EAI self-carriage				
Countries				
United States	Ref	-	-	-
Canada	4.58	2.36	1.67; 12.59	0.003
School Type				
Public	Ref	-	-	-
Private	0.79	0.38	0.31; 2.05	0.64
Child age				
≤5 years	Ref	-	-	-
>5 years	3.70	1.86	1.38; 9.93	0.009

Note: Household income (USD) included as a covariate. Abbreviations: EAI, epinephrine autoinjector; DepVar, dependent variable; OR, odds ratio; SE, standard error; 95%CI, 95% confidence interval.

**Table 4:** Logistic regression predicting the existence of an in-school anaphylaxis protocol.

	OR	SE	95%CI	*p*-Value
Countries				
United States	Ref	-	-	-
Canada	8.98	9.56	1.11; 72.42	0.04
School Type				
Public	Ref	-	-	-
Private	2.64	1.35	0.97; 7.22	0.06
Child age				
≤5 years	Ref	-	-	-
>5 years	3.88	3.03	0.84; 17.97	0.08

Note: Household income (USD) included as a covariate. Abbreviations: OR, odds ratio; SE, standard error; 95%CI, 95% confidence interval.

**Table 5:** Logistic regression predicting restrictions on certain food allergens being sent to school and restrictions on food allergens in specific areas of the school.

	OR	SE	95%CI	*p*-Value
DepVar: Restrictions on food allergens being sent to school				
Countries				
United States	Ref	-	-	-
Canada	2.01	1.34	0.54; 7.43	0.29
School Type				
Public	Ref	-	-	-
Private	2.64	1.46	0.90; 7.79	0.08
Child age				
≤5 years	Ref	-	-	-
>5 years	0.32	0.23	0.08; 1.31	0.11
DepVar: Restrictions on food allergens in specific areas of the school				
Countries				
United States	Ref	-	-	-
Canada	0.89	0.53	0.27; 2.89	0.84
School Type				
Public	Ref	-	-	-
Private	2.19	1.14	0.79; 6.06	0.13
Child age				
≤5 years	Ref	-	-	-
>5 years	0.25	0.17	0.06; 0.99	0.048

Note: Household income (USD) included as a covariate. Abbreviations: DepVar, dependent variable; OR, odds ratio; SE, standard error; 95%CI, 95% confidence interval.

**Table 6:** Logistic regression predicting stock epinephrine in school.

	OR	SE	95%CI	*p*-Value
Countries				
United States	Ref	-	-	-
Canada	0.79	0.48	0.24; 2.61	0.70
School Type				
Public	Ref	-	-	-
Private	1.90	1.18	0.56; 6.42	0.30
Child age				
≤5 years	Ref	-	-	-
>5 years	1.12	0.75	0.30; 4.21	0.86

Note: Household income (USD) included as a covariate. Abbreviations: OR, odds ratio; SE, standard error; 95%CI, 95% confidence interval.

**Table 7:** Linear regression predicting parents’ comfort with the school’s food allergy policies and the degree to which parents believe the food allergy policies are enforced.

	b	SE	95%CI	*p*-Value
DepVar: Comfort with school’s food allergy policies				
Countries				
United States	Ref	-	-	-
Canada	0.16	0.46	−0.75; 1.06	0.73
School Type				
Public	Ref	-	-	-
Private	1.07	0.39	0.30; 1.84	0.007
Child Age				
≤5 years	Ref	-	-	-
>5 years	0.30	0.50	−0.69; 1.30	0.55
DepVar: Perceived enforcement of school food allergy policies				
Countries				
United States	Ref	-	-	-
Canada	1.01	0.45	0.13; 1.89	0.03
School Type				
Public	Ref	-	-	-
Private	0.42	0.36	−0.29; 1.13	0.24
Child age				
≤5 years	Ref	-	-	-
>5 years	−0.41	0.49	−1.38; 0.57	0.41

Note: Household income (USD) included as a covariate. Abbreviations: b, unstandardized regression coefficient; DepVar, dependent variable; SE, standard error; 95%CI, 95% confidence interval.

## Data Availability

The datasets generated and analyzed during the current study are not publicly available out of respect for participant privacy but can be accessed from the corresponding author upon reasonable request.

## Supplementary Materials

The following supporting information can be downloaded at: https://www.mdpi.com/article/10.3390/nu17121971/s1, File S1: Full survey.

## Abbreviations

b	Unstandardized regression coefficient
CAD	Canadian dollars
EAI	Epinephrine autoinjector
GED	General educational development
IgE	Immunoglobulin E
OR	Odds ratio
SD	Standard deviation
US	United States of America
USD	United States dollars
95%CI	95 percent confidence interval
