# Supplementary material for: An Evaluation of Food Allergy Management Practices in a Sample of Canadian and American Schools
Source: Nutrients. 2025 Jun 10;17(12):1971. doi: 10.3390/nu17121971 (PMC12195637; doi:10.3390/nu17121971)
Supplement: Supplementary file 1 [file nutrients-17-01971-s001.zip › nutrients-3676416-supplementary.pdf]

Supplementary Materials – Full survey

To be completed by parent**/guardian** of child with food allergy.

PART 1: SURVEY ELIGIBILITY QUESTIONS

- Has a doctor or other health practitioner told you that **your child** CURRENTLY has an IgE-mediated food allergy?

IgE-mediated allergic reactions *typically occur within minutes to 2 hours of food ingestion and may include hives, swelling, vomiting, abdominal pain, wheezing, shortness of breath, coughing, throat tightness, trouble breathing/swallowing, feeling faint, and/or anaphylaxis.*

- Yes
- No → if no, end entire survey

2. Which food(s) does your child avoid due to food allergy? **Select all that apply.**
    - Cow's milk
    - Soy
    - Egg
    - Wheat
    - Peanuts
    - Tree nuts (e.g., almond, cashew)
    - Fish (e.g., salmon, tuna)
    - Shellfish (e.g., lobster, shrimp)
    - Sesame
    - None → if no, end entire survey
    - Other, please specify _____________________

PART 2: FOOD ALLERGY MANAGEMENT QUESTIONS

3. How old is **your child** with food allergy?
    - Less than 2 years old → if yes, branch to a
    - Greater than or equal to 2 years old → if yes, branch to b

    a. Please specify age in months ______

    b. Please specify age in years ______

4. Does your child currently attend school/daycare?
    - Yes
    - No → if no, end entire survey
    -

5. Is your child homeschooled?
    - o Yes → if yes, end entire survey
    - o No

6. Where is your child's school located? → if neither US nor Canada selected, end entire survey

    Please select country:

    - o United States
        - If United States, please provide state ____________________
        - If United States, please provide zip code _____________________
    - o Canada
        - If Canada, please select province/territory _____________________
        - If Canada, please provide the first three digits of the school's postal code_______________

7. What is your child's current grade/level in school/daycare?
    - o Daycare/Child care centre (not family-provided care)
    - o Preschool
    - o Pre-Kindergarten
    - o Kindergarten
    - o Grade 1-12 → if yes, branch to a
    - o Other, please specify _____________________

    a. Please specify grade level _______

SCHOOL INFORMATION

8. How **comfortable** are you with the school's/daycare's current food allergy protocols/policies on a scale of 1-10, with 1 being the least comfortable and 10 being the most comfortable?

    o 1 2 3 4 5 6 7 8 9 10

9. How well do you feel like the school's/daycare's current food allergy protocols/policies are **enforced** on a scale of 1-10, with 1 being the least well enforced and 10 being the most well enforced?

    o 1 2 3 4 5 6 7 8 9 10

10. In what type of area is your school located?
    - o Rural or remote
    - o Town/suburban
    - o City/urban
    - o Don't know
    - o Decline to answer
    - o Other, please specify _________

11. What is your school type:

- o Public
- o Private, independent
- o Charter
- o Other, please specify _____________

12. What grade levels are included in your child's school?
    - o Youngest grade level: __________
    - o Oldest grade level: ___________

    Choices from a drop down menu: pre-K/junior kindergarten; kindergarten; grade 1….grade 12, grade 13; other, please specify_____

13. What is the primary teaching language of your child's school?
    - o English
    - o French
    - o Spanish
    - o Other, please specify

14. Is there a school nurse present full-time in your child's school?
    - o No
    - o Yes
    - o Don't know/unsure

SCHOOL FOOD ALLERGY MANAGEMENT STRATEGIES

15. Where do students eat meals/snacks? Check ALL that apply.
    - o Cafeteria, lunchroom, dining hall
    - o Classroom
    - o Other, please explain

16. Are any food allergens restricted from being sent to school?
    - o No
    - o Yes
    - o Don't know

17. Are you aware of a policy in your school (or school division/district) regarding the management of life-threatening allergies?
    - o No (Proceed to Question 23)
    - o Yes, school only
    - o Yes, school division/district only
    - o Yes, both school and school/district division
    - o Don't know (Proceed to Question 23)

18. How are school food allergy policies communicated to students/families? Check ALL that apply.
    - Email to families
    - Email to students
    - Paper mailing to families
    - Paper handout to students
    - Printed in school handbook
    - School website
    - Verbally to families
    - Verbally to students
    - Don't know
    - Other (specify) ______________________________________

19. Are any food allergens <u>restricted from being brought into the school from home</u>?
    - No
    - Yes
    - Don't know

20. Does the <u>cafeteria/lunch room/dining hall</u> have <u>designated allergen-free/allergen-friendly tables, zones or areas</u>?
    - No
    - Yes
    - Don't know

21. Are any food allergens <u>restricted from specific classrooms or other areas of the school</u>?
    - No
    - Yes
    - Don't know

22. Who monitors and enforces the school's food allergy protocols/policies to make sure <u>specific food allergens</u> are <u>NOT brought into areas</u> where they are not allowed? Check ALL that apply.
    - Not applicable (there are no restrictions on specific food allergens in the school)
    - Nobody
    - Cafeteria staff
    - Lunch aides
    - Parents
    - School nurses
    - Teachers
    - Students WITH food allergies
    - Students WITHOUT food allergies
    - Don't know
    - Other, please explain ______________________________________

23. How does the monitor make sure specific food allergens are not brought into areas where they are not allowed? Check ALL that apply.
   - o Not applicable (there are no restrictions on specific food allergens in the school)
   - o Ask parents not to send food containing specific food allergens to school
   - o Ask students WITH FOOD ALLERGIES if their food contains specific food allergens
   - o Ask students WITHOUT FOOD ALLERGIES if their food contains specific food allergens
   - o Check appearance of food (i.e., does food look like it has specific food allergens in/on it)
   - o Check smell of food (i.e., does food smell like it has specific food allergens in it)
   - o Read food ingredient labels
   - o Don't know
   - o Other, please explain ______________________________________

24. Does the school have recommended protocols regarding handwashing for students?
   - o No
   - o Yes
   - o Don't know

25. When is handwashing recommended for students? Check ALL that apply.
   - o Not applicable (no specific handwashing protocols)
   - o On arrival to school
   - o Before meals/snacks
   - o After meals/snacks
   - o Other (specify) ______________________________________

26. What is recommended that students use to wash their hands? Check ALL that apply.
   - o Not applicable (no specific handwashing protocols)
   - o Hand sanitizer
   - o Soap and water
   - o Water only (no soap)
   - o Water-based hand/baby wipes
   - o Don't know
   - o Other (specify) ______________________________________

EPINEPHRINE AUTOINJECTOR ACCESS

27. Where is students' epinephrine stored? Check ALL that apply.
   - o Classroom
   - o Health office
   - o Main office or other administrative office
   - o Students permitted to self-carry
   - o Don't know

- Other (specify) ___________________

28. Does the school have stock/undesignated epinephrine available for emergency use?
    - No
    - Yes
    - Don't know

29. Which school personnel are trained in epinephrine administration? Check ALL that apply.
    - Administration
    - Cafeteria staff
    - Lunch aides
    - School nurses
    - Teachers
    - Don't know
    - Other (specify) _____________________________________

30. Does your school have a protocol in place for anaphylaxis management?
    - No
    - Yes
    - Don't know

<u>BULLYING</u>

Bullying is any unwanted aggressive behavior by another youth or group of youths that involves an observed or perceived power imbalance and is repeated multiple times or is highly likely to be repeated. Examples of food allergy-related bullying may include throwing food at a child with food allergies, damaging the belongings of a child with food allergies, calling a child names because of their food allergies, or spreading harmful rumors about a child with food allergies.

31. Is there formal training for <u>students</u> on preventing and addressing bullying?
    - No
    - Yes
    - Don't know

YOUR CHILD'S INFORMATION –

32. Child's identified gender?
    - Boy
    - Girl
    - Non-binary

33. Which of the following best describes your child?
    - Asian
    - Black or African American

- Hispanic
- Indigenous
- Hawaiian or other Pacific Islander
- White
- Multi-Race
- Other, please specify ______________________

YOUR INFORMATION

34. What is your relationship to the child you are answering this survey about?
    - Mother
    - Father
    - Other, please specify ________

35. Your identified gender?
    - Woman
    - Man
    - Non-binary

36. Which of the following best describes you?
    - Asian
    - Black or African American
    - Hispanic
    - Indigenous
    - Hawaiian or Pacific Islander
    - White
    - Multi-Race
    - Other, please specify ______________________

37. What is your annual household income (in CAD/USD)?
    - Less than $20,000
    - $20,000 to $34,999
    - $35,000 to $49,999
    - $50,000 to $74,999
    - $75,000 to $99,999
    - $100,000 to $149,999
    - $150,000 to $199,999
    - $200,000 or more

Here, we will apply branch logic for Canadian or American dollars, based on participants' responses to Question 5, country of residence.

38. What is your highest education level?
    - Did not finish elementary/grade school
    - Elementary/grade school
    - High school degree
    - GED or other high school equivalency certificate
    - Junior/community college degree
    - Undergraduate degree (4-year college degree)
    - Graduate school degree
